# PHLPP2 as a novel metastatic and prognostic biomarker in non‐small cell lung cancer patients

**DOI:** 10.1111/1759-7714.13196

**Published:** 2019-09-30

**Authors:** Hongmei Wang, Ruixue Gu, Fanglin Tian, Yuechao Liu, Weina Fan, Guiqin Xue, Li Cai, Ying Xing

**Affiliations:** ^1^ Department of Pathology Harbin Medical University Cancer Hospital Harbin China; ^2^ The Fourth Department of Medical Oncology Harbin Medical University Cancer Hospital Harbin China; ^3^ General Surgical Department The Fifth Hospital of Daqing Daqing China

**Keywords:** Metastasis, non‐small cell lung cancer, PHLPP2, prognosis

## Abstract

**Background:**

PH domain and leucine‐rich repeat protein phosphatase 2 (PHLPP2) has been reported to be a potent tumor suppressor in many human cancers. However, PHLPP2 has not been fully researched as a putative clinical prognostic biomarker of lung cancer.

**Methods:**

The Cancer Genome Atlas (TCGA) and Gene Expression Omnibus (GEO) databases including data on 1383 non‐small cell lung cancer (NSCLC) patients were used to determine PHLPP2 expression. PHLPP2 expression was then examined by immunohistochemistry, and its clinical significance analyzed in 134 NSCLC patients, including 73 patients with adenocarcinoma and 81 with squamous cell carcinoma.

**Results:**

We found PHLPP2 expression to be less pronounced in NSCLC tissue samples than that in nontumoral lung tissues according to data taken from TCGA and GEO datasets; this outcome was further validated by immunohistochemistry assay. The low PHLPP2 expression level was found to be associated with the presence of lymph node metastasis (*P* = 0.003). Importantly, PHLPP2 was found to be an independent indicator of prognosis for overall (hazard ratio [HR] = 0.520, 95% confidence interval [Cl] = 0.327–0.827; *P* = 0.006) and disease‐free survival (HR = 0.489, 95% Cl = 0.308–0.775; *P* = 0.002) in patients with surgically‐resected NSCLC by multivariate analysis.

**Conclusion:**

Taken together, our findings show that PHLPP2 is a robust clinical marker for NSCLC survival and could serve as a potential therapeutic target.

## Introduction

Lung cancer, one of the most commonly diagnosed malignancies, has an incidence of 11.6% among all new cancer cases and currently accounts for 18.4% of cancer‐related mortality worldwide.^1^ Non‐small cell lung cancer (NSCLC) accounts for a striking 87% of all lung cancer cases with a five‐year survival rate of 21% after diagnosis.^2^ While various treatments such as surgery, chemotherapy, radiotherapy and immunotherapy have improved the prognosis of NSCLC to some extent, the risk of relapse remains high, highlighting the need to identify oncogenic and tumor suppressive genes related to patient prognosis as molecular biomarkers and to develop therapeutic targets.^3^

PHLPP2, a pleckstrin homology domain leucine‐rich repeat protein phosphatases (PHLPP) isozyme, catalyzes the dephosphorylation of AGC kinases including Akt.^4^ Maintaining balanced levels of PHLPP2 expression is essential in preventing pathologies, as changes in steady‐state levels of PHLPP2 are correlated with many diseases including diabetes, hepatic steatosis and cancer.^5^, ^6^ Recently, many studies have shown that PHLPP2 expression is ubiquitously lost in multiple cancers and plays a key role in a wide range of biological processes such as cancer cell proliferation, metastasis, autophagy and apoptosis.^6^, ^7^, ^8^, ^9^, ^10^, ^11^, ^12^

PHLPP2 regulates several cellular pathways in cancer.^13^ Notably, direct dephosphorylation by PHLPP2 inactivates pro‐oncogenic AGC kinases such as Akt, S6K, and PKC and activates pro‐apoptotic kinases such as Mst1.^14^, ^15^, ^16^, ^17^ In addition, PHLPP2 can regulate the epigenome by suppressing histone acetylation and phosphorylation to reduce the gene expression of epidermal growth factor receptor (EGFR), an oncogenic driver of tumor progression.^18^ The essential role of PHLPP2 in promoting the autophagic degradation of MMP2 protein and bladder cancer invasion was recently identified.^19^ In reference to NSCLC, studies of the tumor suppressive effects and functional mechanisms of PHLPP2 are ongoing.^13^ PHLPP2 downregulation was reported to promote lung carcinogenesis by inducing inflammatory tumor necrosis factor alpha (TNFα).^20^ Contactin‐1 contributes to the malignant behavior of lung cancer cells by reducing PHLPP2 expression.^21^ By targeting the 3′ UTR of PHLPP2, miR‐205 and miR‐141 frequently were upregulated and drive malignant phenotypes in NSCLC.^22^, ^23^ However, the diagnostic and prognostic significance of PHLPP2 in NSCLC has not been fully studied.

Therefore, this study aimed to assess protein levels of PHLPP2 in NSCLC tissue samples and found a correlation between PHLPP2 and clinicopathological indicators of NSCLC. In conclusion, our data show that elevated PHLPP2 levels may predict negative lymph node metastasis and the favorable survival of patients with NSCLC.

## Methods

### Patients and tissue specimens

In this study, samples of 134 human NSCLC tissues and 30 normal lung tissues were fixed in formalin and then embedded in paraffin for immunohistochemistry after surgical removal between January 2009 and December 2009 at the Harbin Medical University Cancer Hospital. Patients who had received neoadjuvant therapy or radiotherapy prior to surgery were not enrolled in this work. Follow‐up data for NSCLC patients expired on 30 March 2019 or on the date of death. Clinicopathological information for these patients is given in Table 1. A pathologist provided diagnosis and executed histological typing and tumor staging following the World Health Organization Consensus Classification and Staging System for lung cancer. Our study was approved by the Harbin Medical University Institute Research Medical Ethics Committee.

**Table 1:** Association between PHLPP2 expression and clinicopathological characteristics in 134 NSCLC patients

		PHLPP2 expression		
	All patients	High (%)	Low (%)	
Variable	(*n* = 134)	(*n* = 47)	(*n* = 87)	*P*‐value
Gender				
Male	88	34 (72.3)	54 (62.1)	0.232
Female	46	13 (27.7)	33 (37.9)	–
Age (years)				
<60	90	28 (59.6)	62 (71.3)	0.169
≥60	44	19 (40.4)	25 (28.7)	–
Differentiation				
Well	4	2(44.7)	2(44.8)	0.838†
Moderate	65	23(48.9)	42(48.3)	–
Poor	65	22(6.4)	43(6.9)	–
pTNM stage				
I	72	31 (66.0)	41 (47.1)	0.096
II	23	7 (14.9)	16 (18.4)	–
III	39	9 (19.1)	30 (34.5)	–
pT classification				
T1	42	17 (36.2)	25 (28.7)	0.215†
T2	83	25 (53.2)	58 (66.7)	–
T3/4	9	5 (10.6)	4 (4.6)	–
Lymph node metastasis				
Present	83	37 (78.7)	46 (52.9)	0.003*
Absent	51	10 (21.3)	41 (47.1)	–

* *P* < 0.05 was considered statistically significant.

† The Fisher's exact test.

NSCLC, non‐small cell lung cancer; pT, pathological tumor; pTNM, pathological tumor node metastasis.

### Immunohistochemistry (IHC)

IHC experimental procedures were performed as previously described.^24^ The paraffin‐embedded tissue blocks were sectioned into 4 μm thick portions for IHC. These sections were then deparaffinized and rehydrated. The samples were immersed in 3% H_2_O_2_ solution to block endogenous peroxidase activity before antigen retrieval with citrate buffer (PH 6.0). The sections were then incubated with an anti‐PHLPP2 antibody (1:100 dilution, 25 244‐1‐AP, ProteinTech) at 4°C overnight, after which the slides were incubated with a corresponding rabbit secondary antibody at 37°C. The anti‐PHLPP2 antibody is specific to the peptide sequence of PHLPP2 protein “LDLQHNALTRLPDTLFSKALNLRYLNASANSLESLPSACTGEESLSMLQLLYLTNNLLTDQCIPVLVGHLHLRILHLANNQLQTFPASKLNKLEQLEELNLSGNKLKTIPTTIANCKRLHTLVAHSNNISIFPEILQLPQIQFVDLSCNDLTEILIPEALPATLQDLDLTGNTNLVLEHKTLDIFSHITTLKIDQKPLPTTDSTVTS” encoded by BC129927 (https://www.ncbi.nlm.nih.gov/nuccore/BC129927). Finally, PHLPP2 expression was scored by two pathologists using the following criteria: (i) The percentage of immunopositive cells: 0 (0%), one (0%–10%), two (11%–50%), three (51%–70%), and four (≥71%) and (ii) the intensity of staining: 0 (negative staining), 1 (mild staining), two (moderate staining), three (intense staining). Staining scores collected from the two scoring systems were summed to obtain a final point score of 0 to 7 where 0–3 points denoted low PHLPP2 expression while 4–7 points denoted high PHLPP2 expression.

### Bioinformatics analysis

We used web‐based tools available through GEPIA (Gene Expression Profiling Interactive Analysis, http://gepia.cancer-pku.cn/) based on the TCGA database to detect PHLPP2 mRNA levels in various tumors. NSCLC genome‐wide gene profiles were downloaded from TCGA (https://tcga-data.nci.nih.gov/). Gene expression datasets GSE81089, GSE40419 and GSE19804 were obtained from the publicly accessible GEO (http://www.ncbi.nlm.nih.gov/geo/) database. The Kaplan‐Meier Plotter (http://kmplot.com/analysis/) online tool was used to test the predictive significance of PHLPP2 expression.

### Statistical analysis

We used the SPSS 21.0 statistical software program (Chicago, IL, USA) to analyze the data. Distinctions between the two groups were analyzed with a χ^2^ test. A Kaplan‐Meier analysis was performed to analyze the survival time using a log‐rank test. Using the Cox proportional hazards model, the independent prognostic factors of OS and DFS were analyzed by multivariate analysis. *P*‐value <0.05 was regarded as statistically significant.

## Results

### PHLPP2 exhibited downregulated expression in human NSCLC tissues

**Figure 1:**
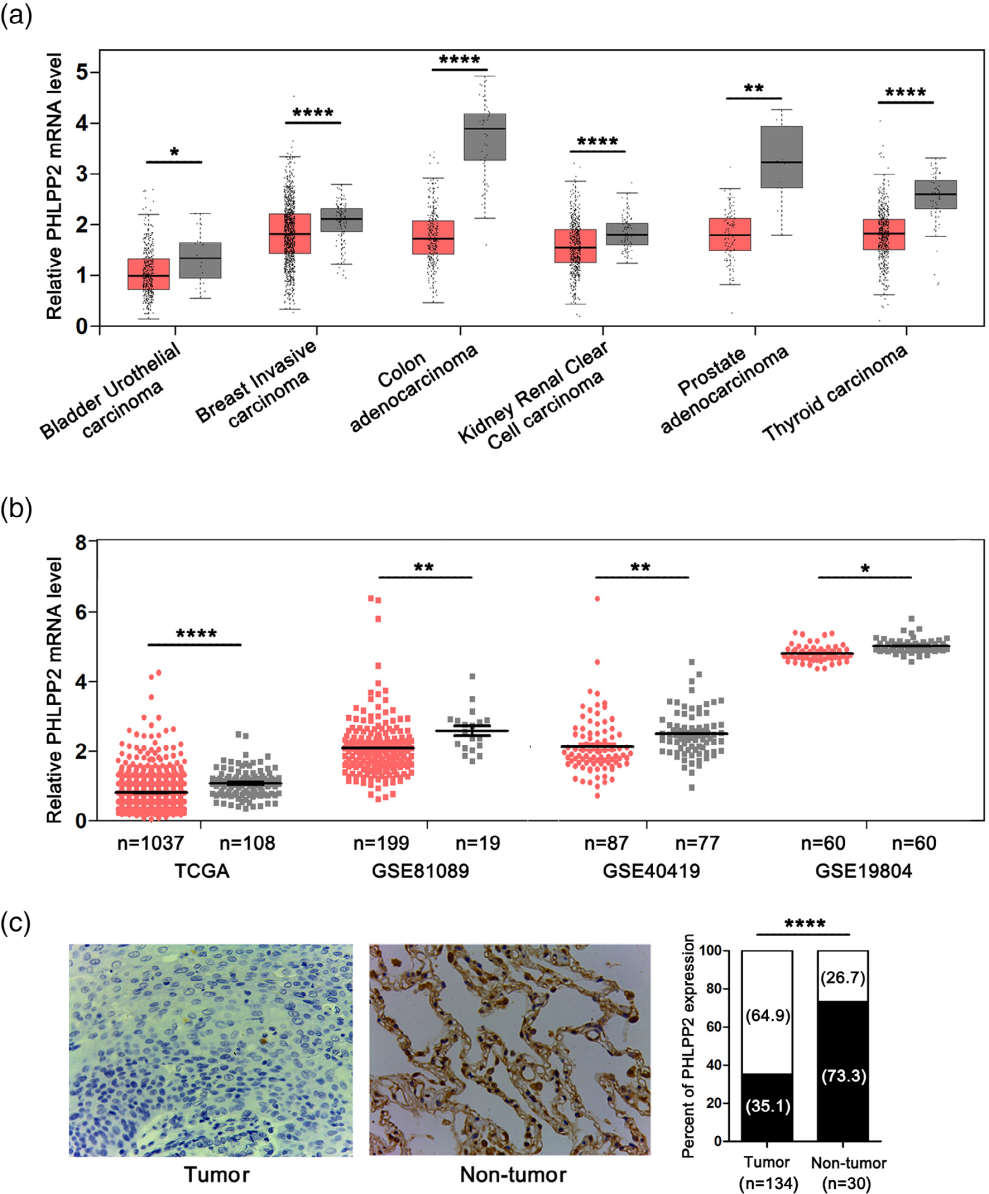
PHLPP2 expression in human NSCLC tissues. (**a**) Relative mRNA levels of PHLPP2 in different tumors were analyzed by GEPIA. (**b**) The expression levels of PHLPP2 mRNA in NSCLC and nontumoral lung tissues were analyzed using TCGA and GEO databases. (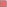) Tumor and (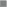) Nontumor. (**c**) Representative immunostaining images for PHLPP2 expression in 134 human NSCLC tissues (Tumor) and 30 normal lung tissues (Nontumor). (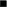) High and (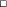) low. Histogram showing percentages of PHLPP2 expression for tumor and nontumor tissues. ****P < 0.0001, **P < 0.01, *P < 0.05.

GEPIA was used to detect PHLPP2 mRNA levels in different carcinomas.^25^ We first determined that PHLPP2 was significantly downregulated in kidney renal clear cell carcinoma and thyroid carcinoma tissues (Fig 1a). Consistent with previous findings for bladder urothelial carcinoma, breast invasive carcinoma, colon adenocarcinoma and prostate adenocarcinoma, PHLPP2 levels were significantly lower in the tumor tissues than those in non‐tumor tissues (Fig 1a).

In NSCLC, PHLPP2 was also found to be downregulated in tumor as opposed to what was observed in nontumor from TCGA and GSE81089, GSE40419, and GSE19804 datasets (Fig 1b). The results demonstrated that PHLPP2 expression levels in the NSCLC tissues were lower than those observed in the nontumoral lung tissues (Fig 1c).

### Correlation between PHLPP2 expression and clinicopathologic characteristics in NSCLC tissue samples

**Figure 2:**
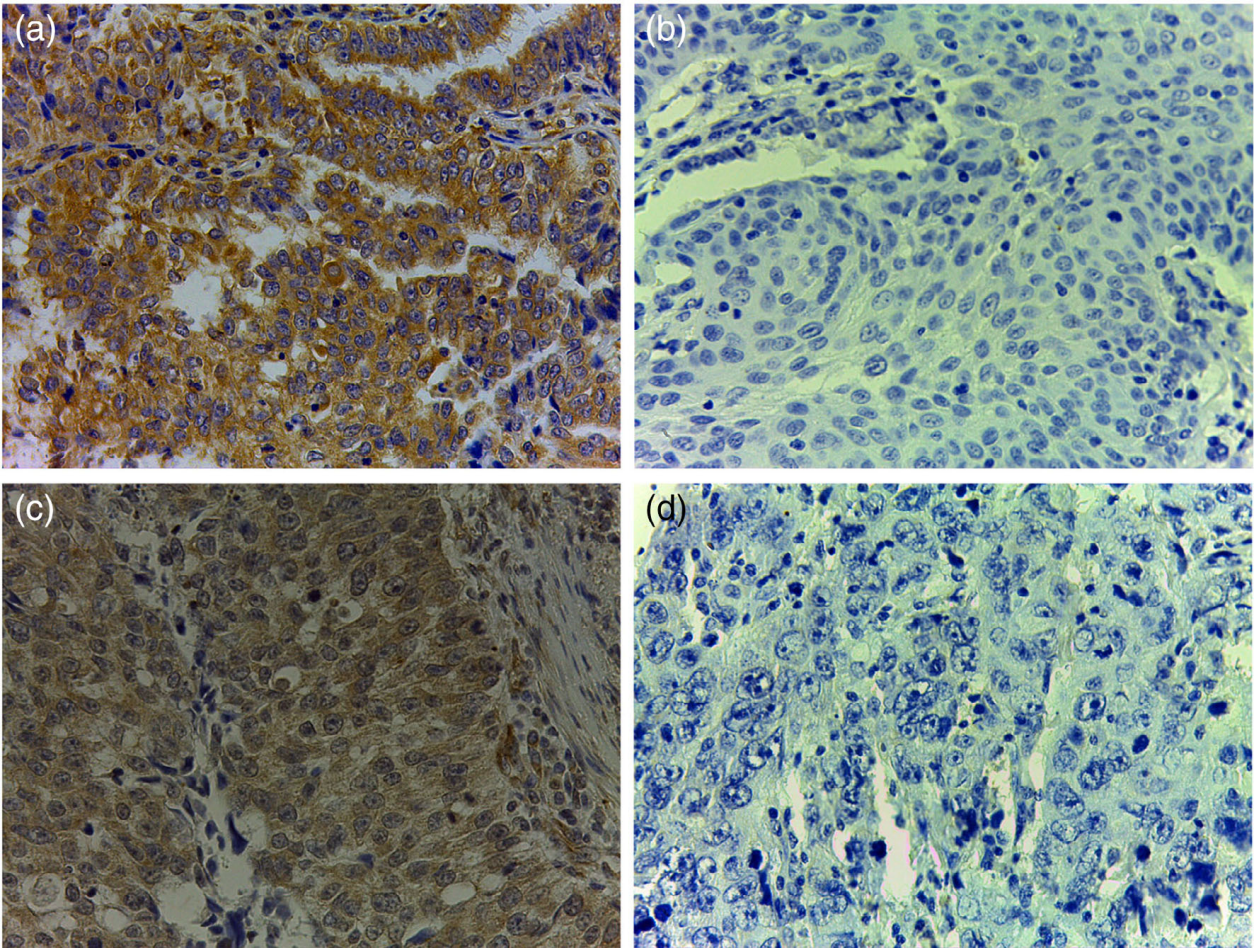
Representative immunohistochemical staining photographs of PHLPP2. (**a**) High and (**b**) low levels of PHLPP2 protein expression in adenocarcinoma. (**c**) High and (**d**) low levels of PHLPP2 expression in squamous cell carcinoma.

Negative control and positive control of PHLPP2 staining was shown in Figure S1. Negative or weak levels of PHLPP2 immunoreactivity were observed in NSCLC tissue samples (64.9%), and PHLPP2 was clearly localized in the cytoplasmic and nuclear compartments of tumor cells (Figs 1c, 2). High and low PHLPP2 expression levels in ADC and SCC tissues by immunohistochemical staining were shown in Figure 2.

The association between PHLPP2 expression and diverse clinicopathological factors are illustrated in Table 1. Low PHLPP2 expression was found to be significantly correlated with the presence of lymph node metastasis (*P* = 0.003). However, no strong associations were observed between PHLPP2 and gender, age, differentiation, pTNM stage and pT classification.

### Low PHLPP2 protein levels predicted poor survival of NSCLC

**Figure 3:**
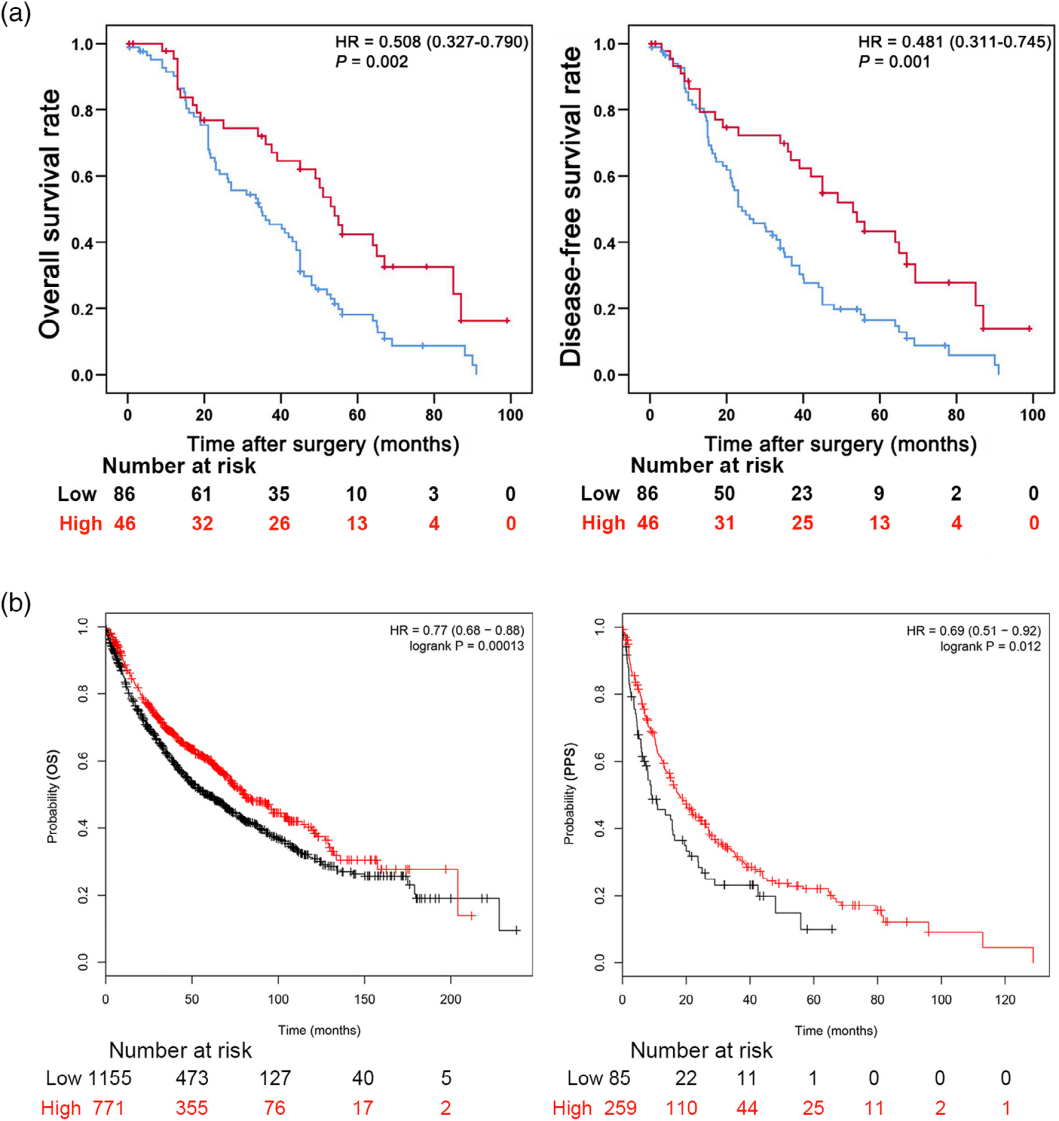
High PHLPP2 expression correlated with favorable survival outcomes in NSCLC patients. (**a**) Kaplan‐Meier analysis of overall (left) and disease‐free survival (right) for high and low PHLPP2 expression levels. (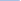) Low‐PHLPP2 and (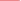) high‐PHLPP2. (**b**) Kaplan‐Meier survival curves of OS and PPS comparing high and low PHLPP2 expression estimates of NSCLC patient survival probability determined from the Kaplan‐Meier plotter database (213407_at). Expression (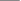) low and (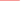) high. * *P* < 0.05 was considered statistically significant.

The survival curves of OS (HR 0.508, 95% CI 0.327–0.790; *P* = 0.002) and DFS (HR 0.481, 95% CI 0.311–0.745; *P* = 0.001) are shown in Figure 3a. Furthermore, the Kaplan‐Meier plotter online tool was used to validate the effect of PHLPP2 on lung cancer survival (*n* = 1926).^26^ The results consistently show that patients with elevated levels of PHLPP2 expression had longer OS (*P* = 0.00013) and post progression survival (PPS) (*P* = 0.012) than those with low PHLPP2 expression levels (Fig 3b). Using the web‐based tools in The Human Protein Atlas (https://www.proteinatlas.org) based on TCGA, low PHLPP2 expression predicts a poor prognosis in ADC (Fig S2).

Furthermore, the results of univariate analysis demonstrated that advanced pTNM stage, positive lymph node metastasis and low PHLPP2 expression were the key factors leading to poorer OS in patients with NSCLC. Multivariate analysis results showed that pTNM stage (HR 1.953, 95% CI 1.368–2.789; *P* < 0.001) and PHLPP2 expression (HR 0.520, 95% CI 0.327–0.827; *P* = 0.006) were independent prognostic factors for OS. The results of univariate analysis of DFS were similar to the findings observed in OS. In our multivariate analysis, pTNM stage (HR 1.892, 95% CI 1.326–2.700; *P* < 0.001) and PHLPP2 expression (HR 0.489, 95% CI 0.308–0.775; *P* = 0.002) were independent predictors for DFS, as shown in Table 2.

**Table 2:** Univariate and multivariate analyses of OS and DFS

	OS			DFS		
	Univariate analysis	Multivariate analysis		Univariate analysis	Multivariate analysis	
Variable	*P*‐value	HR (95% CI)	*P*‐value	*P*‐value	HR (95% CI)	*P*‐value
Age		–	–	–	–	–
< 60		–	–	–	–	–
≥ 60	0.453	–	–	0.610	–	–
Gender		–	–	–	–	–
Female		–	–	–	–	–
Male	0.401	–	–	0.358	–	–
Differentiation		–	–	–	–	–
Good		–	–	–	–	–
Moderate		–	–	–	–	–
Poor	0.053	–	–	0.073	–	–
pT stage		–	–	–	–	–
I		–	–	–	–	–
II		–	–	–	–	–
III/IV	0.109	–	–	0.067	–	–
pTNM stage		–	–	–	–	–
I		–	–	–	–	–
II		–	–	–	–	–
III	<0.001*	1.953 (1.368 to 2.789)	<0.001*	<0.001*	1.892 (1.326 to 2.700)	<0.001*
Lymph node metastasis		–	–	–	–	–
Present		–	–	–	–	–
Absent	0.003*	0.646 (0.336 to 1.244)	0.191	0.002*	0.730 (0.384 to 1.390)	0.339
PHLPP2 expression		–	–	–	–	–
Low		–	–	–	–	–
High	0.003*	0.520 (0.327 to 0.827)	0.006*	0.001*	0.489(0.308 to 0.775)	0.002*

* *P* < 0.05 was regarded as statistically significant.

CI, confidence interval; DFS, disease‐free survival; HR, hazard ratio; NSCLC, non‐small cell lung cancer; OS, overall survival; pT, pathological tumor; pTNM, pathological tumor node metastasis.

## Discussion

The present study illustrates the clinical significance of PHLPP2 in NSCLC. Our data showed that PHLPP2 expression in protein levels in NSCLC tissue specimens are downregulated relative to nontumoral lung tissues. We also found that PHLPP2 expression is deregulated in kidney renal clear cell carcinoma and thyroid carcinoma. Previous studies show decreased expressions of PHLPP2 in several types of cancer.^27^, ^28^, ^29^, ^30^, ^31^ In agreement with our findings, PHLPP2 was lost or decreased in most colon and pancreatic tumor tissues, strongly supporting its tumor suppressive role.^30^, ^31^

Researchers have made efforts to identify novel and important metastatic and prognostic biomarkers to guide research and provide therapeutic targets.^16^, ^32^, ^33^ Another interesting finding was that we found that low PHLPP2 expression levels were associated with positive lymph node metastasis. Moreover, the present study suggested that overexpression of PHLPP2 as an effective molecular biomarker is associated with favorable prognosis for NSCLC. Consistently, low PHLPP2 expression was significantly related to the advanced tumor clinical stage, poor differentiation and positive lymph node metastasis in hypopharyngeal squamous cell carcinoma.^28^ Although there was a trend of the correlation between low PHLPP2 expression and advanced pTNM stage in our study, there was no statistical significance. As this study of lung cancer involved a limited number of patients, it is essential to determine the tumor suppressive role of PHLPP2 as a promising prognostic biomarker based on investigations of larger samples.

However, for 75 advanced lung adenocarcinoma patients receiving EGFR tyrosine kinase inhibitor (EGFR‐TKI) treatment, there was no significant correlation between PHLPP2 protein expression and any clinicopathological characteristics reported by Xie *et al*.^34^ The inconsistent results might be due to discrepancies in different techniques of detection, scoring methods of PHLPP2 expression, unequal cutoff points, sample size, and the treatment of EGFR‐TKI for patients after surgery between these two studies. Xie *et al*. showed that the high expression rate of PHLPP2 reached 61.3% based on the intensity of staining, while in the present study we found 35% of cases with high PHLPP2 expression based on the percentage of immunopositive cells and the intensity of staining.^34^ How to standardize the technology and criteria on PHLPP2 detection is the key point to evaluate its clinical significance. Some miRNAs reported to target the 3′‐untranslated region (3′‐UTR) of PHLPP2, including miR‐27a, miR‐205 and miR‐93, were found to be significantly and positively correlated with lymph node metastasis and poor survival, indicating that PHLPP2 can be defined as an important molecular marker for cancer patients to prognosticate spreading to lymph nodes and prognosis.^23^, ^35^, ^36^, ^37^, ^38^

Our IHC analysis results show that PHLPP2 plays a critical role in tumor cell metastasis. Numerous findings have provided supporting evidence of the tumor suppressive roles of PHLPP2.^17^, ^19^, ^35^, ^39^, ^40^ Peng *et al*. revealed that PHLPP2 promotes MMP2 degradation to inhibit bladder cancer invasion *via* p62‐mediated autophagy.^19^ PHLPP2 has been reported to induce epithelial‐mesenchymal transition (EMT), an important step of tumor metastasis, through an Akt/GSK3β dependent pathway.^35^ Moreover, PHLPP2 suppresses the invasive growth and migration of colorectal cancer cells by negatively regulating EMT and RAF1.^17^

Researchers have made efforts to identify several important cancer biomarkers to guide research and provide therapeutic targets.^32^ The present study suggested that PHLPP2 is an effective molecular biomarker for NSCLC diagnosis. It has consistently been reported that lower PHLPP2 expression levels might predict poor prognosis in hypopharyngeal squamous cell carcinomas.^28^ However, for 75 advanced lung adenocarcinoma patients receiving EGFR tyrosine kinase inhibitor (EGFR‐TKI) treatment, it was PHLPP1 rather than PHLPP2 protein expression levels that were found to be negatively associated with poor outcomes, showing that PHLPP2 has no effect on acquired EGFR‐TKI resistance.^34^ As previous works and our studies of lung cancer involved a limited number of patients, it is essential to determine the tumor suppressive role of PHLPP2 as a promising prognostic biomarker based on investigations of larger samples.

As an identified tumor suppressive gene, multiple lines of evidence reveal the role of PHLPP2 in tumor progression.^7^, ^8^, ^9^, ^13^, ^23^, ^29^, ^41^, ^42^, ^43^ PHLPP2 is involved in the inhibition of malignant behaviors of cancer attributed to the suppression of prosurvival signaling such as through the PI3K/Akt signaling pathway.^13^, ^29^ In addition, PHLPP2 can dephosphorylate and stabilize the Myc oncogene to suppress the progression of prostate cancer.^42^ A recent study reported on a novel molecular cascade of PHLPP2/CREB/miR‐302d that mediates cell cycle progression and anchorage‐independent growth.^41^ Therefore, the identification of PHLPP2 as an effective prognostic biomarker is reasonable.

Strenuous efforts have been made to understand how expression of PHLPP2 itself is regulated. PHLPP2 mRNA translation is regulated by miRNA, the mammalian target of rapamycin complex 1 (mTORC1) and ubiquitinating enzymes.^13^ Many different miRNAs target the 3′‐UTR of PHLPP2 in various cancers.^23^, ^35^, ^38^ Knockdown of key components of the mTORC1 complex results in decreased translation of PHLPP mRNA.^44^ Intriguingly, a recent study suggests a role of free Raptor, which is normally associated with the mTORC1 complex, interacts and binds PHLPP2, resulting in PHLPP2 protein stabilization and reduced signaling through Akt.^45^ Moreover, potassium channel tetramerization domain containing 17 (KCTD17) plays a role in the regulation of PHLPP2 protein stability by targeting to PHLPP2 for ubiquitin‐mediated degradation.^5^

In summary, this study shows that high levels of PHLPP2 expression predict better survival outcomes and that PHLPP2 serves as a promising candidate target for NSCLC. Further studies will continue to shed light on the functions and mechanisms of PHLPP2 as a crucial tumor suppressor in NSCLC. Our study presents knowledge that will prove central to the development of novel therapies targeting PHLPP2.

## Disclosure

The authors have no competing interests to declare.

## Supporting information


**Figure S1** Negative control and positive control of PHLPP2 staining. (**a**) Negative staining of lung cancer. (**b**) Positive control for osteosarcoma.

**Figure S2** Kaplan–Meier curves showing survival for ADC patients with high and low PHLPP2 expression from TCGA database.
