# Supplementary figures and images for: PHLPP2 as a novel metastatic and prognostic biomarker in non‐small cell lung cancer patients
Source: Thorac Cancer. 2019 Sep 30;10(11):2124–32. doi: 10.1111/1759-7714.13196 (PMC6825916; doi:10.1111/1759-7714.13196)

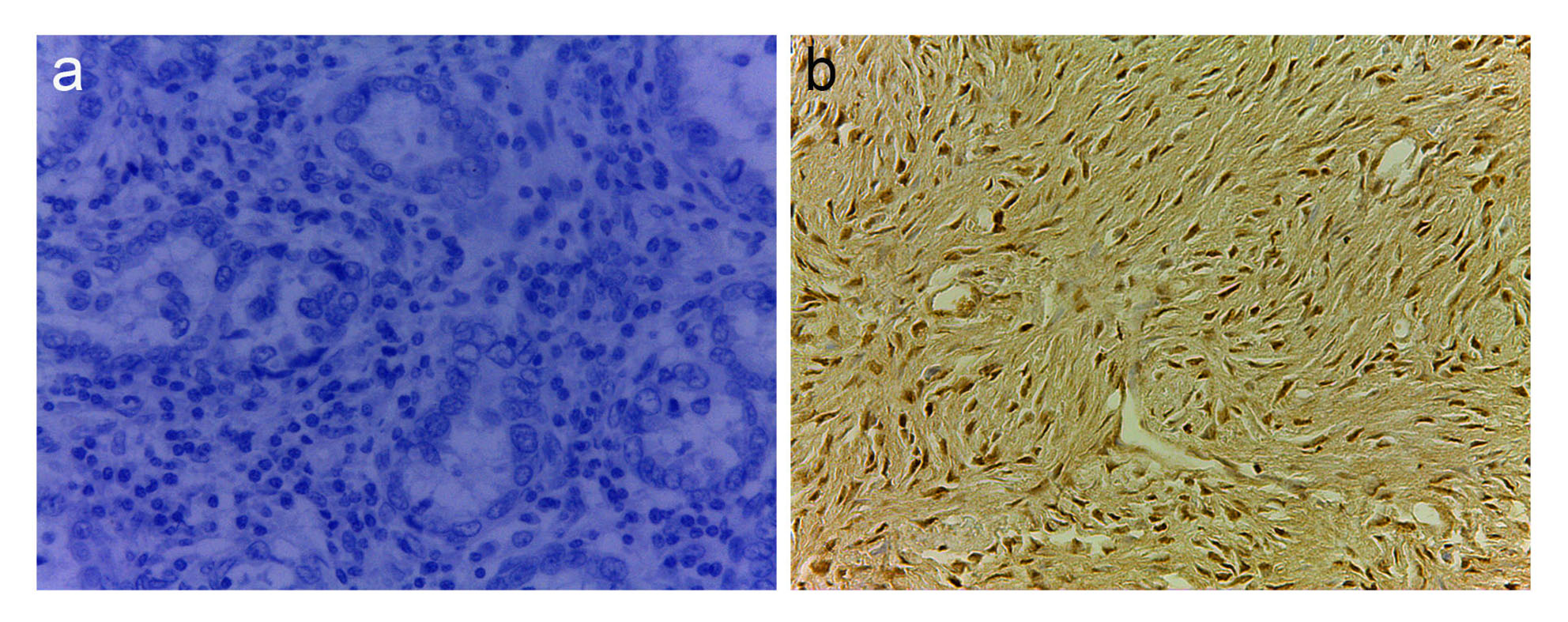

Supplement: Supplementary file 1 — Figure S1 Negative control and positive control of PHLPP2 staining. (a) Negative staining of lung cancer. (b) Positive control for osteosarcoma. [file TCA-10-2124-s001.jpg]

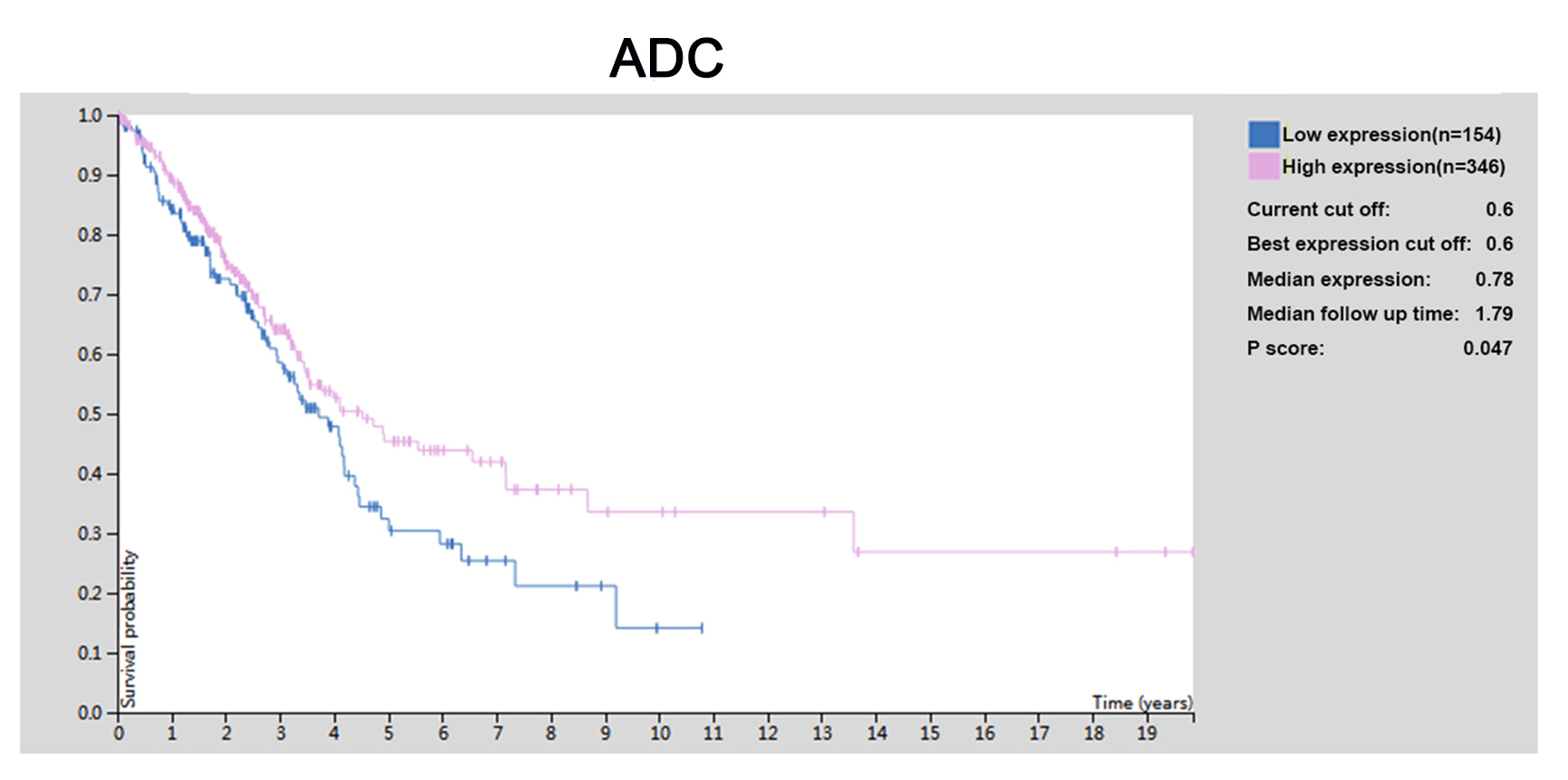

Supplement: Supplementary file 2 — Figure S2 Kaplan–Meier curves showing survival for ADC patients with high and low PHLPP2 expression from TCGA database. [file TCA-10-2124-s002.jpg]
